# Supplementary material for: A conformational fingerprint for amyloidogenic light chains
Source: eLife. 2025 Mar 3;13:RP102002. doi: 10.7554/eLife.102002 (PMC11875538; doi:10.7554/eLife.102002)
Supplement: Supplementary file 1. — (a) Pairwise sequence identity (above diagonal) and similarity (below diagonal) for the six systems under study. On the diagonal is reported the germline identified by igBLAST using the IGMT database. (b) HDX-MS data summary. [file elife-102002-supp1.docx]

**Supplementary file 1a.** Pairwise sequence identity (above diagonal) and similarity (below diagonal) for the 6 systems under study. On the diagonal is reported the germline identified by igBLAST using the IGMT database.

|  | H3 | H7 | H18 | AL55 | M7 | M10 |
| --- | --- | --- | --- | --- | --- | --- |
| H3 | ***IGLV1-44*01*** | 179/216 (82.9%) | 168/216 (77.8%) | 171/218 (78.4%) | 163/216 (75.5%) | 172/217 (79.3%) |
| H7 | 194/216 (89.8%) | ***IGLV1-51*01*** | 169/214 (79.0%) | 168/218 (77.1%) | 165/214 (77.1%) | 169/217 (77.9%) |
| H18 | 183/216 (84.7%) | 182/214 (85.0%) | ***IGLV3-19*01*** | 166/218 (76.1%) | **196/214 (91.6%)** | 171/217 (78.8%) |
| AL55 | 190/218 (87.2%) | 188/218 (86.2%) | 184/218 (84.4%) | ***IGLV6-57*02*** | **164/218 (75.2%)** | 175/218 (80.3%) |
| M7 | 181/216 (83.8%) | 179/214 (83.6%) | **204/214 (95.3%)** | **182/218 (83.5%)** | ***IGLV3-19*01*** | 169/217 (77.9%) |
| M10 | 196/217 (90.3%) | 185/217 (85.3%) | 186/217 (85.7%) | 188/218 (86.2%) | 183/217 (84.3%) | ***IGLV2-14*03*** |

**Supplementary file 1b.** HDX-MS data summary.

| **Datasets** | **H3** | **H7** | **AL55** | **M10** |
| --- | --- | --- | --- | --- |
| **HDX reaction details** | 1X Phosphate buffer saline in D_2_O (pD 7.0), 25°C | | | |
| **HDX time course (min)** | 0, 0.5, 1, 10, 30, 120, and 240 | | | |
| **Back exchange (mean/IQR)** | ND | | | |
| **No. of peptides** | 61 | 50 | 57 | 62 |
| **Sequence coverage (%)** | 98.6 | 92.5 | 98.6 | 99.1 |
| **Average peptide length/ Redundancy** | 13.6/4.16 | 15.8/4.01 | 14.3/4.06 | 15.1/4.69 |
| **Replicates (technical)** | 3 | 3 | 3 | 3 |
| **Repeatability (average SD)** | 0.04 Da | 0.04 Da | 0.04 Da | 0.05 Da |

**Legends for Datasets**

**Dataset S1 (separate file).**

DOI: 10.5281/zenodo.12731283, https://dx.doi.org/10.5281/zenodo.12731283

Molecular dynamics simulation trajectories and associated statistical weights.
